# Molecular Mechanisms and Current Pharmacotherapy of Peyronie’s Disease: A Review

**DOI:** 10.3389/fphar.2021.643641

**Published:** 2021-05-20

**Authors:** Fuxun Zhang, Feng Qin, Jiuhong Yuan

**Affiliations:** ^1^ Andrology Laboratory, West China Hospital, Sichuan University, Chengdu, China; ^2^ Department of Urology, West China Hospital, Sichuan University, Chengdu, China

**Keywords:** peyronie’s disease, mechanisms, pharmacotherapy, treatment, wound-healing

## Abstract

Peyronie’s disease (PD) is a localized fibrotic lesion of the penis that has adverse effects on men’s health. In this review, we summarized the molecular mechanisms and pharmacotherapies of PD. A literature search was conducted using PubMed and Cochrane Library during 2001–2020. Although no oral or topical medication demonstrated efficacy in monotherapy of PD, several intralesional medications have yielded promising results. Currently, the effective strategy in management of PD should be combined modality therapy, including but not limited to pharmacotherapy, mechanical therapy, and psychotherapy. Meanwhile, basic research is still necessary to facilitate the development of novel and more reliable treatments. In future, more attention should be given simultaneously to epigenetic changes, inflammatory cytokines, the abnormal wound-healing process, and profibrotic and anti-fibrotic factors to provide more options for this refractory disease.

## Introduction

In the spectrum of fibrotic conditions, penile fibrosis is characterized by disproportionate accumulation of collagen components in the tunica albuginea, penile corpora cavernosa, and corpus spongiosum, termed as Peyronie’s disease (PD), corporal fibrosis, and urethral stricture respectively (Garaffa et al., 2013; Milenkovic et al., 2019a). Among them, PD is a localized penile lesion possibly associated with micro-trauma during intercourse and an abnormal wound-healing process (Gonzalez-Cadavid and Rajfer, 2005). As a progressive fibrotic disease, PD often results in local pain, penile deformity, difficult penetrative intercourse, erectile dysfunction (ED), mental diseases and relationship difficulties, which have several adverse impacts on the life quality of males, sometime seriously (Gaffney and Kashanian, 2020). It is reported that the incidence of PD might be 22.4 to 25.7 per 100,000 men, with the average age of patients being 55 years (Herati and Pastuszak, 2016). However, the prevalence of PD is likely underestimated due to under-reporting bias from those never seeking treatment (Chung et al., 2020). Although the mechanisms of PD are not fully understood several molecular pathological changes of the PD plaque have been recognized recently (Graziottin, 2015; Paulis et al., 2017). Currently, available satisfactory treatments for PD are limited, and in certain situations non-surgical medication might be an important alternative therapeutic approache to PD. This review summarizes the current perspectives on molecular mechanisms and available pharmacotherapies of PD, in order to provide reasonable therapy regimens for clinicians.

## Pathophysiology

In most situations, fibrotic disorders are strongly associated with ceaseless damage of cells and tissue caused by various injurious factors, including mechanical, infectious, and autoimmune elements. As a localized and chronic fibrotic disease of the tunica albuginea, PD is mainly caused by repetitive trauma in tunica albuginea related to intercourse, resulting in penile deformity, veno-occlusive dysfunctional ED, depression, and damaged relationships (Nelson et al., 2008; Garaffa et al., 2013). There are two hypotheses regarding the pathogenesis of PD. The first one states that the tunica albuginea was separated by repeated trauma at the inner and outer layer, especially at the dorsomedial aspect where the intercavernous septum is formed (Gonzalez-Cadavid and Rajfer, 2005; Graziottin, 2015). The second hypothesis suggests that this separation might occur between sinusoidal tissue and the inner layer of tunica albuginea, subsequently forming micro hematoma in the connective tissue sleeve between tunica albuginea and corpus cavernosum (Space of Smith) (Milenkovic et al., 2019b). The lesion site involving the dorsomedial aspect of tunica albuginea and the distinctive extension to the corpus cavernosum provides evidence for these hypotheses.

Generally, the inflammatory response and extra-cellular matrix (ECM) deposition induced by various traumatic factors constitute the normal reparative process. However, this process might transform into chronic fibrogenesis if the injurious agents are not removed in time, leading to gradual tissue remodeling (Cannito et al., 2017). Scanning electron microscope shows excessive collagen I/III deposition, decreased normal collagen architecture, and disordered elastic fibers in PD plaques (Ostrowski et al., 2016). The abnormal scarring process in tunica albuginea reduces penile elasticity and stretch, leading to curvature and deformation of penis.

PD is divided into the acute phase, characterized by painful erections and a worsening penile curvature that is contraindicated to surgery, which occurs over 12–24 months, and the chronic phase, characterized by stable malformation and fibrotic plaque (Garaffa et al., 2013; Milenkovic et al., 2020). Meanwhile, there has been no association of PD with penile trauma reported in previous studies, indicating that not only traumatic factors but some unknown elements may have played a role in the development of PD (Zargooshi, 2004; Gabrielsen, 2020). Although the etiology of PD is unclear, it is proposed that genetic susceptibility and several molecular mechanisms might be responsible for this disease.

## Genetic Predisposition

Significantly, 22% of PD cases concur with Dupuytren, a fibrotic disorder implicated in palmar fascia (Nugteren et al., 2011). Considering the correlation between Dupuytren contracture and PD, genetic predisposition might modulate the wound-healing and fibrotic process in PD (Gonzalez-Cadavid and Rajfer, 2005). However, a previous study serologically typed 154 consecutive PD patients for human leukocyte antigen (HLA) and demonstrated that no significant association between PD and HLA antigens exist (Hauck et al., 2003). Recently, the decreased gene expression of insulin-like growth factor 1 (IGF1) isoforms in tunica albuginea was found using samples from PD plaques of 24 patients, suggesting that IGF1 gene may participate in the development of PD (Thomas et al., 2016). Recently, a study performed an in-depth analysis using next-generation RNA sequencing and found many differentially expressed genes. It is reported that kappa-light-chain-enhancer of activated B cells (NF-κB) and signal transducer and activator of transcription proteins (STAT)-signaling might be important to the fibrotic process of PD (Milenkovic et al., 2019c). Therefore, although the genetic role in PD has not been studied intensively, current data may suggest that various unknown genetic changes contribute to the pathogenesis of PD, providing potential targets for genetic therapy.

## Molecular Mechanisms

Fibrosis, a reactive and wound-healing process, is characterized by excessive accumulation of fibrotic connective tissue. In the wound-healing process, various insults trigger an inflammatory response and facilitate the migration of innate immune cells to the sites of injury, resulting in the release of various biological mediators which stimulate the phenotype activation of fibroblast to myofibroblast and promote ECM production (Wynn, 2008).

Myofibroblasts, a critical part of the normal wound-healing process, are considered as the main cause of fibrosis in PD (Darby and Hewitson, 2007). After the tissue heals, the activated matrix-producing myofibroblasts can end up in apoptosis or return to quiescent phenotype, however, the wound-healing event would evolve into fibrosis if any procedure in this process was hindered. Currently, the major source of myofibroblasts is still controversial (Mack and Yanagita, 2015). These key cells may originate from stem cells in the tunica albuginea or other cell lines which obtained ECM-synthesizing phenotype after exposure to transforming growth factor β1 (TGFβ1), such as local fibroblasts, smooth muscle cells (SMCs), and endothelial cells. Therefore, blocking the phenotype transformation of other cells into myofibroblasts and modulating stem cells in the tunica albuginea might provide promising strategies for treatment of PD (Nolazco et al., 2008; Mack and Yanagita, 2015).

Another important culprit in PD is the activation of platelet and coagulation system induced by the trauma of tunica albuginea (Gonzalez-Cadavid and Rajfer, 2005). A wide range of growth factors, including platelet derived growth factor (PDGF) - a potent mitogen and chemotactic molecule for fibrogenic cells, are released after activation of platelets (Kazlauskas, 2017). Together with other mediators, PDGF in turn promotes the metabolic effects of TGFβ up-regulation, activity of myofibroblasts, and recruitment of fibrotic cells, enhancing the ECM deposition and fibrotic plaque formation (Lucattelli et al., 2008). Moreover, increased expression of PDGF α/β receptors were observed in many fibrotic diseases (Klinkhammer et al., 2018). In line with other fibrotic conditions, elevated levels of PDGF α/β and their receptors have been demonstrated in animal models of PD (Lucattelli et al., 2008). Therefore, blocking PDGF signaling pathway might be a promising strategy in treatment for PD and other fibrotic diseases.

Earlier experimental studies have shown that reactive oxygen species (ROS) and plasminogen activator inhibitor 1 (PAI-1) induced by initial insults penile tissues are remarkably increased in fibrotic plaques and then result in oxidative stress (Davila et al., 2003). With the increasement of these profibrotic agents and oxidative stress, the level of nitric oxide (NO) synthesized by inducible nitric oxide synthase (iNOS) isoform elevated simultaneously both in human and animal plaques, which could quench the ROS and inhibit the collagen synthesis and plaque formation together with its product cGMP (Vernet et al., 2002; Davila et al., 2003). Previous studies on the iNOS knockout mouse have confirmed the anti-fibrotic role of iNOS and emphasized the importance of iNOS in protecting SMCs in the penile corpora cavernosa (Ferrini et al., 2010). Moreover, an animal study also found that the levels of iNOS and hypoxia-inducible factor-1 (HIF-1) rise in PD-like lesions (Krakhotkin et al., 2020). Thus, the inhibition of profibrotic gene expression or activity might be an exciting addition to PD therapy in future.

If the injurious events were not removed in the repair process, the inflammation might persist and several immune cells would be recruited to release various enzymes and cytokines, resulting in more lasting tissue damage, loss of parenchymal cells, and release of profibrotic mediators. In this setting, TGFβ1 is probably the primary pro-fibrogenic factor in the formation of fibrotic plaques (Davila et al., 2003). There are three active members in the TGF-β family, TGF-β1, TGF-β2, and TGF-β3, which possess similar biological functions *in vitro* (Gressner and Weiskirchen, 2006). Among them, TGF-β1 is the pivotal member in wound-healing reparation and collagen accumulation (Gressner and Weiskirchen, 2006). A large variety of studies using experimental models have demonstrated that the injection of recombinant TGFβ1 into tunica albuginea or sub-tunica could result in the formation of PD-like plaques over the course of several weeks, meanwhile blocking the signaling pathway of TGFβ1 could inhibit this pathological courses (Gonzalez-Cadavid and Rajfer, 2009; Chung et al., 2011). Furthermore, the fibrotic plaque is a consistent pathological process involving balancing mechanisms and interaction of molecular signals and cellular activation (Qian et al., 2004). In here, small mothers against decapentaplegic proteins (SMADs) showed multiple effects on TGF-β signaling pathway in PD. It is reported that phosphorylation of SMAD2/3 increased TGF-β1-induced fibroblasts from PD plaques, and that SMAD7 may have an antagonistic role on TGF-β1 signaling and subsequently limit the response of fibroblasts to TGF-β1 in PD (Herati and Pastuszak, 2016). Therefore, the potential loss or under-expression of SMAD7 might remove a key blockade in the inflammatory cascade and facilitate PD.

On the other hand, it is reported that TGFβ1 is a possible driver of Rho-associated coiled-coil protein kinase (ROCK) (Milenkovic et al., 2019d). Activation and up-regulation of ROCK have been examined in the corpora cavernosa following neuropraxia (Sezen et al., 2014). The potential pro-fibrotic mechanism of ROCK in penile fibrosis may be mediated by the myofibroblast and TGFβ1 (Chitaley et al., 2001). Several studies indicated that TGF-β1 signaling pathway proceeding in PD plaque might be associated with nuclear translocation of Rho/RhoA-mediated Yes-associated protein/transcriptional coactivator with PDZ-binding motif (YAP/TAZ), and ROCK inhibitor could induce the apoptosis of fibroblasts and attenuate collagen synthesis in tunica albuginea of PD patients (Knipe et al., 2015; Milenkovic et al., 2019a). Thus, the pro-fibrotic ingredients such as TGFβ1, oxidative stress, and ROCK are indicative of penile fibrotic process, and inhibiting these factors by anti-fibrotic agents such as iNOS, NO, and cGMP could ameliorate or block this process.

Fibrotic conditions often involve an imbalance between deposition and degradation of ECM in the proliferation and remodeling phase. Abnormal degradation of ECM components seems to be the most important factor in fibrogenesis in PD. Matrix metalloproteinases (MMPs), a zinc-dependent protease family, is induced by several inflammatory cytokines, such as tumor necrosis factor-α (TNF-α), interleukin-1 (IL-1), and NO (Del Carlo et al., 2008). The MMPs may possess a double-sided nature, namely anti-fibrotic and pro-fibrotic effects, and involve complicated interactions with tissue-inhibitors of metalloproteinases (TIMPs). In scar formation, MMPs with anti-fibrotic property are down-regulated, while the expression of TIMPs in myofibroblasts and macrophages are up-regulated (Prihadi et al., 2018; Milenkovic et al., 2019b). Moreover, IL-1β is considered to be a stimulus of MMP-1, MMP-3, MMP-8, MMP-9, MMP-10, and MMP-13 in fibroblasts derived from PD plaques. Meanwhile, TIMPs 1 to 4 were detectable in tunica albuginea from PD patients (Del Carlo et al., 2008). Additionally, several studies have shown that MMPs might have certain impacts on cellular behavior, such as gene expression, proliferation, and apoptosis, which affect fibrosis in turn (Roderfeld, 2018). These findings indicated that an imbalance between MMPs and TIMPs might provide a fibrotic co-factor for PD. Here, driving expression of MMPs and decreasing the expression of TIMPs, might be a promising target to break down the fibrotic plaque.

In summary, the molecular mechanism of PD is a complicated pathological process involved with abnormal signaling pathway, sophisticated interaction in pro/anti-fibrotic cells, and imbalance between accumulation and degradation of collagen. In all, persistent tissue injury provokes chronic inflammation and an increase of pro-fibrotic factors, such as TGF-β1, ROS, PAI-1, ROCK, TNF, and TIMPs, which induce the phenotype transformation of precursor cells to myofibroblasts. With excessive deposition of ECM, the formation of fibrotic plaque appears to be inevitable, leading to penile deformation and malfunction. Therefore, it is plausible to speculate that regaining the balance between the protective and detrimental mechanisms is a mainstay in the pharmacotherapy of PD.

## Pharmacotherapy for PD

**FIGURE 1:**
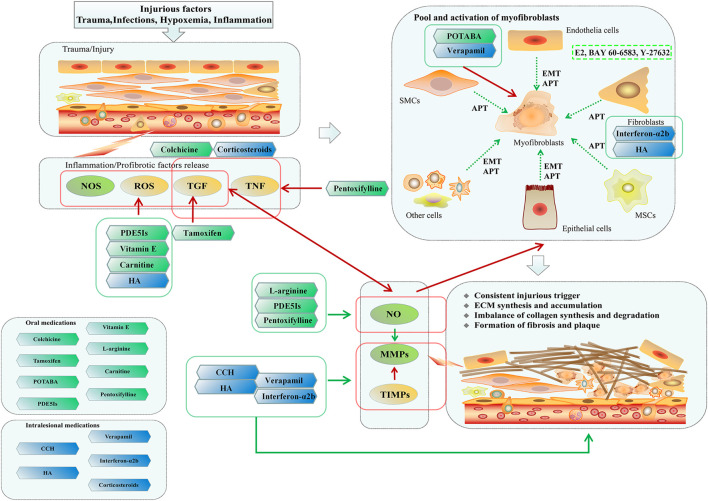
Possible mechanisms of Peyronie's disease and pharmacotherapeutic therapies. Due to the injurious factors were not removed timely, consistent injurious trigger result in chronic inflammation and the release of profibrotic factors, such as TGF, ROS, and TNF. Furthermore, these events induce phenotype transformation from potential precursors to myofibroblasts, and subsequently imbalance of collagen synthesis and degradation. Finally, the persistent accumulation of ECM leads the formation of penile fibrosis and plaque, causing penile deformation and dysfunction. In clinical practices, pharmacotherapy remains an important part of combination regimen for Peyronie's disease. Vitamin E, Carnitine, PDE5Is, L-arginine and HA were reported to be beneficial to quench the ROS. Tamoxifen might have the anti-fibrotic effects by inhibiting secretion of TGF-β1. Pentoxifylline has anti-inflammatory and anti-fibrotic role by increasing NO and inhibiting the TGF-β1 and TNF. Colchicine could inhibit the inflammation and increase collagenase activity. POTABA and verapamil exhibits the anti-fibrotic effect by suppressing the activation of myofibroblasts. Interferon-α2b and HA could reduce the proliferation of fibroblasts, and might have the role of immune modulation. Corticosteroids could inhibit the inflammation and the activity of phospholipase A2. CCH, HA, verapamil, and Interferon-α2b have the advantages of decreasing deposition of ECM, of which, CCH and HA could directly degrade the collagen, whilst verapamil and Interferon-α2b reduce the collage synthesis and increase collagenase activity. As potential novel drugs, E2, BAY 60-6583 and Y-27632 could suppress the TGFβ1-induced myofibroblast transformation.

Currently, surgery is still the gold-standard treatment for PD (Wymer et al., 2019). Although surgery could correct the penile deformity and provide functional recovery to a certain extent, non-surgical medication, especially for those unwilling or unable to accept surgery, remains an important part of combination regimen for attenuating the fibrotic process, reducing penile deformity and improving intercourse. Moreover, surgery is often a difficult choice for patients, and the risk of surgical complications is also a crucial issue even for an experienced surgeon (Reddy et al., 2018). Thus, pharmacotherapy and even empirical regimen still a very important position in management of PD (Figure 1; Table 1).

**TABLE 1:** Current and potential pharmacotherapy for Peyronie’s disease.

Agent	References	Route	Mechanisms	Adverse effects	Recommendation
Vitamin E	Levine and Burnett (2013)	Oral	Anti-oxidant property and immune modulation, quench the ROS, reduce the deposition of collagen in tunica albuginea	Diarrhea, headache, nausea, vomiting, dizziness, possible cerebrovascular accidents	Negative
	Paulis et al. (2013)				
	Safarinejad et al. (2007)				
L-arginine	Valente (2003)	Oral	Substrate of NOS, accelerate the NO production, reduce the collage synthesis and deposition in Peyronie’s plaques	Diarrhea, gastrointestinal spasm, nausea, seizures, vomiting, possible acceleration of herpesvirus replication, administration with caution in patients with medical history of herpes	Combination therapy for PD
	Paulis et al. (2017)				
	Rajfer et al. (2006)				
Carnitine	Safarinejad et al. (2007)	Oral	Anti-oxidant property, quench the ROS, reduce the deposition of collagen in tunica albuginea	Diarrhea, gastrointestinal spasm, nausea, seizures, vomiting	Negative
	Calò et al. (2006)				
	Biagiotti and Cavallini (2001)				
Pentoxifylline	Tsambarlis and Levine (2019)	Oral	Nonspecific phosphodiesterase inhibitor, anti-inflammatory and anti-fibrotic effect by inhibiting secretion of TGF-β and TNF, reduce the collage synthesis and deposition in Peyronie’s plaques	Angina, diarrhea, dizziness, headache, indigestion, leukopenia, myelosuppression, nausea, vomiting, thrombocytopenia, possible hypotension needs monitor	Combination therapy for PD

	Singh et al. (2015)				
	Albersen et al. (2011)				
Colchicine		Oral	Anti-inflammatory effect, increase collagenase activity, reduce the collage synthesis and deposition	Diarrhea, myelosuppression, nausea, vomiting	Negative
	Kadioglu (2003)				
	Prieto Castro (2003)				
Tamoxifen	Carthy et al. (2015)	Oral	Anti-fibrotic effects by inhibiting secretion of TGF-β1 via non-SMAD pathway, block TGF-receptors	Alopecia, decreased libido, erectile dysfunction, headaches, pancytopenia, retinopathy, thrombosis and embolism, nausea, vomiting	A potential component in combination therapy
	Brandt et al. (2014), van der Bilt et al. (2016)				
	Teloken et al. (1999)				
POTABA	Teloken and Katz (2007)	Oral	Anti-fibrotic effect by inhibiting secretion of fibroblast glycosaminoglycan and stabilizing activity of serotonin-monoamine oxidase	Anxiety, anorexia, fever, hypoglycemic episodes, headache, nausea, skin rash	Negative
	Weidner et al. (2005)				
	Roy and Carrier (2008)				
PDE5Is	Valente et al. (2003), Tsambarlis and Levine (2019)	Oral	Increase the cGMP level, reduce profibrotic factors and myofibroblasts, quench the ROS, reduce the collage synthesis and deposition, preserve SMCs	Dizziness, diarrhea, flushing, headache, indigestion, rhinitis, skin rash, administration with caution in patients with medical history of cardiovascular events	Combination therapy for PD or corporal fibrosis
	Bjekic et al. (2006), Ferrini et al., (2006a)				
	Palmieri et al. (2012)				
	Ozturk et al. (2014), Montorsi et al. (2016)				
Verapamil	Han et al. (2016), Levine et al. (2002)	Intralesional/Topical	Anti-fibrotic effect by inhibiting transference of calcium into fibroblasts and stimulating phenotype transformation of fibroblast into non-synthetic status, reduce the collage synthesis and deposition, increase collagenase activity	Ecchymosis, headache, nausea, penile pain, no cardiovascular side effects observed	Topical verapamil is not recommended, intralesional verapamil could be a part of a combination therapy for PD
	Chung et al. (2013), Shirazi et al. (2009)				
	Shindel et al. (2008), Randhawa and Shukla (2019)				
Interferon-α2b	Hellstrom et al. (2006),	Intralesional	Immune modulation, inhibit the fibroblasts proliferation, reduce the collagen synthesis and deposition, increase collagenase activity	Ecchymosis, fevers, influenza-like symptoms, sinusitis	Combination therapy or monotherapy for PD
	Campbell and Alzubaidi (2017)				
	Stewart et al. (2015)				
Corticosteroids	Cipollone et al. (1998), Demey et al. (2006)	Intralesional	Anti-inflammatory effect, immune suppression, inhibit phospholipase A_2_	Local tissue fibrosis, skin atrophy and thinning, rare systemic side effects, possible increasement of difficulty for surgery	Negative
	Lamprakopoulos et al. (2000)				
	Russell et al. (2007)				
HA	Litwiniuk et al. (2016), Gennaro et al. (2015), Favilla et al. (2017), Cocci et al. (2020)	Intralesional	Anti-inflammatory effect, anti-oxidant effect, quench the ROS, immunosuppression	Sporadic ecchymosis, rare major side effects	Combination therapy or monotherapy for acute phase of PD
CCH	Gelbard et al. (2013), Lipshultz et al. (2015), Hellstrom et al. (2019), Wymer et al. (2019)	Intralesional	Anti-fibrotic effect by degrading the collagen and fibrotic plaques	Corporal rupture, ecchymosis, hematoma, penile swelling, pain	Combination therapy or monotherapy for PD
H-100	Twidwell and Levine (2016)	Topical	Compound of nicardipine, superoxide dismutase, and emu oil, similar mechanism as verapamil	Rash at the application site	Potential novel drug
	Tsambarlis and Levine (2019)				
E2	Jiang et al. (2015)	*in vitro*	Inhibit the myofibroblast transformation, decrease the expression of collagen, attenuate the contraction of myofibroblasts	NA	Potential novel drug
BAY 60-6583	Mateus et al. (2018)	*in vitro*	Adenosine receptor A2B agonist, inhibit the myofibroblast transformation in response to TGF-β1	NA	Potential novel drug
Y-27632	Milenkovic et al. 2019b	*in vitro*	A ROCK inhibitor, inhibit the myofibroblast transformation, prevention of YAP/TAZ nuclear translocation	NA	Potential novel drug
	Yamamoto et al. (2012)				
ADSCs	Chamberlain et al. (2007), Dellis and Papatsoris (2017)	Intralesional	Decrease the expression of TIMPs, enhance the expression of MMPs, drive the apoptosis of myofibroblasts, reduce the expression of collagen I and *α* smooth muscle actin, inhibit the Rho/RhoA and SMAD signaling pathway	No report	Could be tested in combination therapy of PD
	Zhang et al. (2010), Gokce et al. (2014)				
	Jiang et al. (2017), Castiglione et al. (2013)				
	Castiglione et al. (2019), Levy et al. (2015)				

Abbreviations: ROS, reactive oxygen species; NOS, nitric oxide synthase; NO, nitric oxide; PD, Peyronie’s disease; TGF, transforming growth factor; TNF, tumor necrosis factor; POTABA, potassium aminobenzoate; PDE5Is, phosphodiesterase type 5 inhibitors; cGMP, cyclic guanosine mono-phosphate; SMCs, smooth muscle cells; HA, hyaluronic acid; CCH, collagenase *clostridium histolyticum*; E2, 17β-estradiol; ADSCs, adipose-derived stem cells; NA, not applicable.

### Oral Medication

Although oral medication requires compliance, it is considered as a comfortable regimen for most patients. Oral treatment is also a relatively inexpensive regimen with few side effects compared with other approaches. However, as an effective drug concentration in local lesions is difficult to reach, the efficacy of oral therapies remains controversial, and high-level evidence is still needed.

### L-Arginine

It is demonstrated that L-arginine could reduce the expression of collagen I, increase NO production, and inhibit ECM synthesis in Peyronie’s fibrotic plaques of animal model (Valente et al., 2003). Although there are no randomized controlled trials (RCTs) performed to evaluate the role of L-arginine in treatment of PD, it shows encouraging efficacy on improving the penile curvature as an agent of combination regimen in a retrospective study (Abern et al., 2012). Moreover, L-arginine, as described in a clinical case report, may attenuate the corporal fibrosis resulting from recalcitrant priapism and therefore could be brought into anti-fibrotic therapy regimen in penile fibrotic disease (Rajfer et al., 2006). Overall, L-arginine might have a positive role in the treatment of PD based on current data, and the authors support its use in combination regimen.

### Pentoxifylline

It is reported that pentoxifylline may reduce the TGFβ1 level in tissue and therefore have an anti-fibrotic role in rats (Raetsch et al., 2002). Based on a meta-analysis with high-level evidence, pentoxifylline could decrease lobular inflammation and improve fibrosis in patients with nonalcoholic steatohepatitis (Singh et al., 2015). Pentoxifylline as a nonspecific phosphodiesterase inhibitor for the treatment of PD is supported by various evidence from basic studies. Particularly, pentoxifylline has shown the properties *in vitro* to attenuate the deposition of collagen in tunica albuginea and reduce the secretion of TNF by T cells that are associated with the pathogenesis of PD (Tsambarlis and Levine, 2019). Meanwhile, pentoxifylline could enhance nerve regeneration and subsequently improve erectile function in rat models of cavernous nerve injury, providing possibility for clinical practices (Albersen et al., 2011). Thus, pentoxifylline could be taken into account as a part of combination treatment for PD, and a medication merits application in corporal fibrosis with ED.

### Tamoxifen

Independent of anti-androgen, tamoxifen serving as an oral agent in treatment of PD relies on its anti-fibrotic effects by inhibiting TGFβ1 via non-SMAD pathway (Carthy et al., 2015). It is reported that tamoxifen has shown anti-fibrotic effects in animal model of periportal hepatic fibrosis and renal fibrosis (Ryu et al., 2009; Dellê et al., 2012). Moreover, the anti-fibrotic role of tamoxifen has been exhibited in several clinical studies which evaluated the mono-therapeutic efficacy of tamoxifen in patients with retroperitoneal fibrosis (Brandt et al., 2014; van der Bilt et al., 2016). Disappointingly, similar studies performed in patients with PD have drawn inconsistent conclusions in which results from a small RCT did not show any significant improvement in the tamoxifen group compared with the placebo group (Teloken et al., 1999). However, a recent study indicated that a combination regimen of tamoxifen and phosphodiesterase type 5 inhibitors might be effective in treating PD based on *in vitro* and *in vivo* disease models (Ilg et al., 2019). In general, although the application of tamoxifen for PD still needs confirmation by clinical trials, the authors suggested that tamoxifen might be a potential component in combination therapy.

### Phosphodiesterase Type 5 Inhibitors

It is reported that phosphodiesterase type 5 inhibitors (PDE5Is) might be a potential cause of PD due to increasing rigidity via oral PDE5Is making the penile deformity more obvious and increasing the susceptibility to penile trauma during intercourse (Bjekic et al., 2006; Tsambarlis and Levine, 2019). However, PDE5Is is a well-researched mechanism that has been widely used for treatment of ED, and has been supported by previous studies for treatment of corporal fibrosis and PD (Ferrini et al., 2006a; Ferrini et al., 2006b). The long-term administration of PDE5Is might inhibit the fibrotic process via decreasing the degradation of cGMP and therefore increase NO downstream signaling (Valente et al., 2003). Moreover, several animal studies have demonstrated that PDE5Is may preserve SMCs and inhibit corporal fibrosis at a genetic level in models with cavernous nerve injury, encouraging the use of PDE5Is in patients after radical prostatectomy (Sirad et al., 2011).

Although amelioration of penile deformity was not examined in an RCT comparing shock wave therapy alone with shock wave therapy plus PDE5Is, other investigators drew the conclusion that continuous therapy of PDE5Is may be a candidate in treatment of PD based on the significant improvement of observational parameters (Palmieri et al., 2012; Ozturk et al., 2014). Additionally, PDE5Is share the characteristics of efficacy and safety in extensive treatment of ED and rehabilitation of postoperative erectile function (Montorsi et al., 2016). According to the above-mentioned animal studies and clinical trials, it is reasonable to take PDE5Is into the combination regimen of penile fibrosis.

### Intralesional Medication

Intralesional injection could deliver the agents into the pathological site with relative high concentration and avoid systemic side effects where possible. Given that fibrotic plaque is similar to mechanical abnormal structure, the appropriate micro-trauma by the injection may be beneficial to plaque remodeling and treatment similar to operative resection (Hellstrom et al., 2006). However, intralesional therapy has some intrinsic limitations and the potential risk of injection-related complications, such as rupture of tunica albuginea, hemorrhage, and hematoma. On the other hand, the injection was superior to oral routes that require more time commitment, patient compliance, and expenditure. Moreover, the efficacy and safety of the intralesional route for patients with plaque calcification and penile ventral curvature are insufficient. Overall, intralesional medications have revealed promising results but must be administrated prudently.

### Verapamil

As a calcium channel blocker used in the treatment of hypertension, verapamil plays an anti-fibrotic role by blocking the transportation of calcium into fibroblasts, inhibiting the transfer of proline into the matrix and stimulating the phenotype transformation of fibroblasts to produce collagenase (Han et al., 2016). Many studies support the intralesional medication of verapamil in treating PD due to possible improvement on clinical features, such as penile curvature, plaque size, and sexual function (Levine et al., 2002; Chung et al., 2013). However, a single-blind RCT that included 80 patients has shown ineffectiveness of intralesional verapamil compared with the control group (Shirazi et al., 2009). Although heterogeneous conclusions were drawn in this respect, intralesional verapamil as one of the most common prescriptions in the United States has exhibited many advantages in the treatment of PD, including mild side effects, lower cost, and the role in stabilizing PD (Shindel et al., 2008; Randhawa and Shukla, 2019). Thus, current evidence suggests that intralesional verapamil might be beneficial to a subset of patients, especially those in acute phase with minimal calcification.

### Interferon-α2b

Interferon is a kind of low molecular weight protein characterized by modulating immune function in anti-proliferative or anti-neoplasm effects (Hellstrom et al., 2006). The application of intralesional interferon α2b (IFNα2b) in treatment of PD is based on that *in vitro* IFNα2b could decrease fibroblasts proliferation, reduce the collagen synthesis and deposition, and increase collagenase activity in fibrotic plaques (Campbell and Alzubaidi, 2017). Meanwhile, a single-blind clinical trial has revealed statistical significance of ameliorating penile deformity using intralesional IFNα2b (Hellstrom et al., 2006). Although some therapeutic benefits appear to exhibit more statistical significance than clinical meaning, the seemingly tiny improvement in these trials is capable of being replicated. Moreover, IFNα2b has the added advantage of treating ventral curvatures for which other intralesional therapies are contraindicated (Stewart et al., 2015). In short, despite the side effects which present as influenza-like symptoms in most cases and are well-tolerated, intralesional IFNα2b could be taken into consideration as an integrated medication in treating PD.

### Corticosteroids

Intralesional corticosteroids were used in the treatment of PD because of its property of inhibiting inflammation. Although corticosteroids were considered as the first intralesional medication in PD, early published studies, including retrospective analyses and RCTs, have had mixed findings (Cipollone et al., 1998; Demey et al., 2006). Moreover, an observational study in 2000 demonstrated that local injection of betamethasone is an effective treatment for PD with low rate of severe complications (Lamprakopoulos et al., 2000). However, the efficacy of intralesional corticosteroids in this study is limited to specific patient groups with medical history less than 12 months as well as plaque size less than 20 mm. Meanwhile, it is proposed that the effective results of intralesional corticosteroids cannot be reproduced (Yafi et al., 2015). On account of the local or systemic adverse effects, increased surgical difficulty from intralesional medication, and insufficiency of high-level evidence, intralesional corticosteroids should not be recommended for treatment of PD (Russell et al., 2007).

### Hyaluronic Acid

Hyaluronic acid (HA) is the main component of ECM and key participant in tissue regeneration. HA has been demonstrated to have multiple roles in the wound-healing process (Litwiniuk et al., 2016). Among them, the effects of immunosuppression and anti-inflammation from high weight hyaluronic molecules of HA form the basis for treatment of PD (Gennaro et al., 2015; Litwiniuk et al., 2016). Recently, a multi-center RCT compared the efficacy of intralesional HA with verapamil in treatment of PD, indicating that HA has greater effectiveness on improvement of penile curvature and patient satisfaction (Favilla et al., 2017). Moreover, a prospective clinical study with a similar purpose demonstrated that intralesional therapy of HA is an effective and reliable option in management of PD in acute phase, and that HA may have a role in amelioration of International Index of Erectile Function (IIEF) (Cocci et al., 2020). Based on this evidence and lower rates of side effects, the authors hold the opinion that HA appears to be advisable in treating PD.

### Collagenase Clostridium Histolyticum

Collagenase *clostridium histolyticum* (CCH), previously used in treating Dupuytren contracture, possesses anti-fibrotic properties by degrading the collagen and is the only FDA - approved intralesional drug for treatment of PD (Wiseman et al., 2019). Two large RCTs, The Investigation for Maximal Peyroni’s Reduction Efficacy and Safety Studies (IMPRESS) I and II, examined the efficacy and safety of intralesional clostridial collagenase in the treatment of PD and obtained high-level evidence (Gelbard et al., 2013). Meanwhile, another study on defined subgroups in IMPRESS I and II confirmed the clinical efficacy of intralesional clostridial collagenase for improvement on penile curvature and PD-related symptoms, and demonstrated that adverse events (AEs) were moderate or mild (Lipshultz et al., 2015). Moreover, a multi-institutional retrospective analysis recently confirmed the safety and efficacy of intralesional clostridial collagenase in treatment of PD patients with low rate of AEs (Hellstrom et al., 2019).

Although patients with penile curvature from 30° to 90° were included in IMPRESS I/II and the deformity were corrected for mean 17° in CCH-treated group, greater overall satisfaction of IIEF was also achieved in treatment group, indicating that the improvement in satisfaction is associated not only with reduced curvature but also objective elements. Besides, penile curvature less than 30° may have no significant impact on intercourse and partnership. Thus, current limitations concerning intralesional collagenase are of a high expenditure, and have an uncertain effect on specific patients with severe curvature and extensive calcification (Wymer et al., 2019). Thus, clostridial collagenase seems to be an effective agent in management of PD with moderate penile curvature. However, clostridial collagenase is not the first-line medication for PD patients with specific plaques and severe deformation, such as painful plaque, calcific plaque, penile curvature more than 90°, and hourglass deformities.

### Topical Medication

Topical approaches for drug delivery for PD were used due to its simplicity, painlessness, and safety. However, the main concern for topical therapies is whether adequate drug concentrations could be accomplished at the targeted site (Martin et al., 2002). Moreover, topical medications may have the risk of producing local skin reactions of the penis. Current topical agents for treatment of PD mainly included calcium channel blockers and calmodulin blockers, such as verapamil, trifluoperazine, magnesium sulfate, and compound H-100 (Twidwell and Levine, 2016). Among them, most agents might have a similar mechanism of action to verapamil. A published pilot study that included 57 patients has shown the efficacy of topical verapamil to ameliorate erectile pain, penile deformity, and plaque size (Fitch et al., 2007). However, this study lacks a control group and objective measurements. As adequate verapamil could not be delivered into the lesion by topical approach, this route is still controversial (Martin et al., 2002).

In recent years, electromotive drug administration (EMDA) as a new delivery approach was developed to increase the concentration of drugs into specific tissues (Mehrsai et al., 2013). Hence, the mechanism of action for drugs administrated by EMDA in PD is no different. Although EMDA gained extensive attention from investigators and might provide potential therapeutic benefit, current evidence does not support its use in the treatment of PD (Greenfield et al., 2007; Mehrsai et al., 2013). In general, further studies are necessary to determine whether topical medication could be an effective option for treatment of PD.

### Potential Novel Drugs of PD

As mentioned above, the phenotype transformation of fibroblast to myofibroblast in tunica albuginea has an important role in the pathogenesis of PD. It is reported that 17β-estradiol (E2) could suppress the TGFβ1-induced myofibroblast transformation and reduce the collagen production by inhibiting the TGF-β1/SMAD and Rho/RhoA signaling pathway (Jiang et al., 2015). In all, E2 seems to be a currently available treatment against this transformation.

On the other hand, the role of adenosine receptors in transformation from fibroblast to myofibroblast in PD was investigated recently. In particular, adenosine receptors (ADOR) A1 and A2B were expressed in both PD plaque-derived cells and tunica-derived cells (Mateus et al., 2018). Among various drugs, BAY 60-6583 as an ADOR A2B agonist was found to significantly inhibit the myofibroblast transformation in response to TGF-β1. Thus, ADOR provides a potential target for treatment of PD and ADOR A2B agonists might be effective for PD in active stage.

Additionally, it has been proposed that ROCK inhibitor could attenuate TGF-β1 signaling and myofibroblast transformation (Yamamoto et al., 2012; Milenkovic et al., 2019c). Meanwhile, Rho/RhoA signaling works partly through nuclear translocation of YAP/TAZ (Knipe et al., 2015). Y-27632, a ROCK inhibitor, coupled with simvastatin has been shown to suppress the TGF-β1-induced myofibroblast transformation by prevention of YAP/TAZ nuclear translocation (Milenkovic et al., 2019d). In this respect, the inhibition of YAP/TAZ nuclear translocation might have anti-fibrotic effects, providing a novel target for PD. Although ROCK-inhibitors appear to be promising for patients with PD at an early stage, it should be noted that the role of a pharmacologic antagonist of ROCK is not restricted to penile tissue, which might incur serious side-effects when systemically used. At present, Fasudil is the only available ROCK-inhibitor in clinical practice used for angina and cerebral vasospasm. This agent is well tolerated with few serious adverse events. Thus, the authors suggest that Fasudil might be a rational drug for treatment of PD.

Most noteworthy, there is an increasing number of emerging therapies for several fibrotic disorders, e.g., pulmonary, liver, and kidney fibrosis (Mora et al., 2017; Milenkovic et al., 2019a). Among them, various signaling pathways as drug targets have been investigated, such as TNF, PDGF, JAK-STAT, PGE2, PDGF, NADPH oxidase, integrin inhibition, mTOR inhibition, and so on. However, not all targets truly offer benefits in treatment of different fibrotic diseases. Therefore, more studies are needed to further confirm the role of these signaling in PD therapy.

### Stem Cell Therapy of PD

Available data has shown that adipose-derived stem cells (ADSCs) could increase the production of growth factors, modulate the ECM, replace the injurious tissue, and diminish inflammation (Chamberlain et al., 2007). In recent years, with rapid development of regenerative medicine, stem cells (SCs) therapy has been tested in treating PD (Dellis and Papatsoris, 2017). Actually, the mechanism of action for ADSCs in therapy of PD remains unknown. It is proposed that ADSCs could migrate to the traumatic sites in response to chemo-attractants and induce products of immunomodulation, inhibiting the inflammatory response to injury and pro-fibrotic process (Zhang et al., 2010). Moreover, it is demonstrated that intralesional allogeneic ADSCs could decrease the expression of TIMPs and enhance the expression of MMPs, reducing the chances of Peyronie’s-like plaque (Gokce et al., 2014). On the other hand, allogeneic ADSCs appear to reduce the expression of collagen I and *α* smooth muscle actin (αSMA) of myofibroblasts in tunica albuginea, and inhibit the Rho/RhoA and SMAD signaling pathway (Jiang et al., 2017). Meanwhile, ADSCs may drive the apoptosis of myofibroblasts and decrease the deposition of collagen by upregulation of MMPs and caspases.

The effects of intralesional human ADSCs on animal model of PD in acute phase was first assessed in 2013 (Castiglione et al., 2013). In this study, ADSCs treatment was proven to significantly improve erectile function and prevent the fibrotic changes in tunica albuginea of PD. This valuable information indicated that SC therapy could be effective for the treatment of PD in acute phase. However, most PD patients seek medical care in stable phase. Hence, the efficacy of SC on chronic PD model deserves further study. A study published in 2019 discussed this issue and has shown that injection of human ADSCs could decrease the expression of collagen III in animal model of PD in chronic phase (Castiglione et al., 2019). Although this evidence cannot be translated into practical applications directly, it may provide a new insight into treatment of PD and subsequently support SC treatment for PD in human study.

Because of these potential limitations, a single-center, phase I, non-randomized study was conducted to evaluate the efficacy and safety of SC therapy for patients with PD using placental matrix–derived mesenchymal SC (PM-MSCs) (Levy et al., 2015). Although this study lacked a control group and enrolled just five patients, the results indicate that SC therapy may be effective for patients with PD. Further, other similar trials enrolling humans are still ongoing.

In a word, despite the lack of qualitative trials with larger samples and long-term follow-ups, SC therapy has revealed the advantage of balancing pro-fibrotic and anti-fibrotic elements. The authors suggest that SC therapy may provide a novel strategy and could be tested in combination therapy of PD. Moreover, it is reported that SC therapy for skin rejuvenation could be conducted by an iontophoretic transdermal transport system in animal model (Ueda, 2014). The authors thus hypothesize that the application of ADSCs by iontophoretic or EMDA system could be considered as a combination therapy for PD.

## Summary

The research on molecular mechanisms and treatment of PD has attracted attention in recent years. Actually, PD shares some similar molecular mechanisms with other fibrotic disorders, which facilitates the development of novel pharmacological experiments and therapies. However, the exploration on a small segment of complicated fibrotic processes in PD is still a mainstream view in current studies. It means that the efficacy of monotherapy could be offset by other signaling pathways. So far, multiple agents with diverse delivery routes have been investigated and applied in treatment of PD. However, the efficacy of these agents is limited.

Although no oral medication in monotherapy has demonstrated reliable results, data from some basic studies appears to support some oral medication. Together with few side effects and low cost, we hold the opinion that taking oral medications, such as L-arginine, pentoxifylline, and PDE5Is, into consideration as a part of combination regimen for PD is reasonable and pragmatic. Meanwhile, no strong evidence supports the use of vitamin E, potassium aminobenzoate, carnitine, and colchicine to date. As the efficacy of a treatment for PD is evaluated partly using subjective PD and ED questionnaires, we hypothesize that some effects of these drugs might be attributed to a placebo effect as opposed to a real response. By contrast, the intralesional therapy used for PD seems to yield better results, meaning clostridial collagenase is considered as an optimal medication. However, clostridial collagenase is very expensive and not suitable for all PD patients. As an alternative drug delivery route, topical medications have not shown promising results and need further evaluation in future trials.

It seems that all fibrotic diseases have the transformation of fibroblast to myofibroblast as a common fundamental. Drugs targeting the inhibition of myofibroblast transformation are gaining increasing attention from researchers. Despite the lack of further confirmation, 17β-estradiol, adenosine receptor A2B agonist, and ROCK inhibitor have shown to be effective for acute PD. Regenerative medicine as a new branch of medical science has developed rapidly in recent years. Several studies explored the anti-fibrotic effects of ADSCs on PD-like plaque and found exciting results. Despite the limited data from human trials, current studies have focused on specific molecular mechanisms and used representative animal models of PD. Meanwhile, the use of SC could redress the balance between pro-fibrotic and anti-fibrotic roles as a whole. Thus, SC therapy has the potential to be integrated into combination treatment of PD.

Taken together, the effective non-surgical treatment for PD should be combination modality, including various pharmacotherapies, mechanical therapies, shock wave therapies, psychotherapies, and so on, which might generate synergy benefits to PD patients. Meanwhile, the basic research on pathogenesis is still necessary to provide more perspectives on therapeutic options. In future, more effective regimens should focus simultaneously on the epigenetic changes, inflammatory cytokines, abnormal wound-healing process, and balance between profibrotic and anti-fibrotic factors in order to provide promising options for this refractory disease. Due to the lack of specific markers used in diagnostic and therapeutic evaluation, treatment for penile fibrotic diseases is still lacking. Thus, it is of equal importance to bring genetic phenotyping and biomarkers into clinical studies of patients with PD. Ultimately, how to regain balance between ECM deposition and degradation might become the cornerstone to attenuate PD and other fibrotic conditions.

## Data Availability

The datasets analyzed during the current study are available from the corresponding author on reasonable request.

## Abbreviations

ROS: reactive oxygen species; NOS: nitric oxide synthase; NO: nitric oxide; TGF: transforming growth factor; TNF: tumor necrosis factor; ECM: extracellular matrix; MMPs: matrix metalloproteinases; TIMPs: tissue-inhibitors of metalloproteinases; EMT: epithelial-mesenchymal transition; APT: activation, proliferation and transformation; SMCs: smooth muscle cells; POTABA: potassium aminobenzoate; PDE5Is: phosphodiesterase type 5 inhibitors; HA: hyaluronic acid; CCH: collagenase clostridium histolyticum; E2: 17β-estradiol.
